# The Shared Ethical Responsibility of Medically and Non-medically Qualified Experts in Human Drug Development Teams

**DOI:** 10.3389/fphar.2018.00843

**Published:** 2018-09-03

**Authors:** Sandor Kerpel-Fronius, Sander Becker, Jane Barrett, Johan Brun, Roberto Carlesi, Anthony Chan, Luis F. Collia, Dominique J. Dubois, Peter Kleist, Greg Koski, Chieko Kurihara, Luis F. Laranjeira, Johanna Schenk, Honorio Silva

**Affiliations:** ^1^Department of Pharmacology and Pharmacotherapy, Semmelweis University, Budapest, Hungary; ^2^Pharmaceutical Medicine, Dover Heights, NSW, Australia; ^3^Pharmaceutical Medicine, Cheshire, United Kingdom; ^4^Life Science, Pfizer, Stockholm, Sweden; ^5^Independent Researcher, Bellagio, Italy; ^6^Pfizer Healthcare Ireland, Dublin, Ireland; ^7^Craveri Pharma, Buenos Aires, Argentina; ^8^PHARMED Post-Graduate Programme in Pharmaceutical Medicine and Medicines Development Sciences, Université Libre de Bruxelles, Brussels, Belgium; ^9^Cantonal Ethics Committee, Zurich, Switzerland; ^10^Alliance for Clinical Research Excellence and Safety, Harvard Medical School, Boston, MA, United States; ^11^Quality Assurance and Audit Office, National Institute of Radiological Sciences, National Institute for Quantum and Radiological Science and Technology, Chiba, Japan; ^12^AMPIF, Medical Department, Eli Lilly & Co., Lisbon, Portugal; ^13^PPH plus GmbH & Co. KG, Hochheim am Main, Germany; ^14^IFAPP Academy, New York, NY, United States

**Keywords:** ethics, ethics committee, medicines development, pharmaceutical medicine, multidisciplinary research

## Abstract

The complexity of developing and applying increasingly sophisticated new medicinal products has led to the participation of many non-medically qualified scientists in multi-disciplinary non-clinical and clinical drug development teams world-wide. In this introductory paper to the “IFAPP International Ethics Framework for Pharmaceutical Physicians and Medicines Development Scientists” it is argued that all members of such multidisciplinary teams must share the scientific and ethical responsibilities since they all influence directly or indirectly both the outcome of the various phases of the medicines development projects and the safety of the research subjects involved. The participating medical practitioner retains the overriding responsibility and the final decision to stop a trial if the well-being of the research subjects is seriously endangered. All the team members should follow the main ethical principles governing human research, the respect for autonomy, justice, beneficence and non-maleficence. Nevertheless, the weighing of these principles might be different under various conditions according to the specialty of the members.

For hundreds of years, treatments based on experience formed a continuum with uncontrolled individual therapeutic trials performed by the treating physicians in the hope of helping their patients. The deep ethical concern of the practicing physicians is expressed with great clarity by William Withering who introduced digitalis into medical practice in the 18th century: “After all, in spite of opinion, prejudice or error, Time will fix the real value upon this discovery, and determine whether I have imposed upon myself and others, or contributed to the benefit of science and mankind.” (Eichhorn and Gheorghiade, 2002).

The major shift toward prospective clinical trials can be traced back to the end of the nineteenth century when major hospitals were founded where trained medical personnel could perform well planned clinical trials. In addition, the creation of medical journals meant that the results could be rapidly communicated to other medical teams working world-wide, creating an international background for establishing common norms for accepted medical practice. Unfortunately, in the rapidly changing medical research environment some physicians performed human experiments which clearly violated the broadly accepted ethical principles of society. During this period the German scientific community, represented by outstanding clinical scientists such as Rudolf Virchow, Robert Koch, Paul Ehrlich, and Emil von Behring made breakthrough contributions to medicine.

It is therefore not surprising that the first regulation of clinical experiments was penned in Germany in 1901 (Erlass der Preussischen Regierung vom Dezember, 1901) in which most of the major ethical issues of clinical research at the time were listed. The complexity of contemporary medical interventions could be relatively easily managed by the clinicians without extensive support of other non-medically qualified experts. Accordingly, it was a reasonable decision by the law makers to place the entire ethical responsibility on the head of the medical team and proclaim that it was the duty of the senior chief physician to evaluate both the scientific and ethical aspects of the research plan and supervise its execution according to Hippocratic Oath governed primarily by the maxim “primum non-nocere.”

In practical terms the responsibility for the safety and well-being of the trial subject means that the ethical responsibility is essentially also the burden of the medical profession. It is explicitly stated in the Declaration of Helsinki: “It is the duty of physicians who are involved in medical research to protect the life, health, dignity, integrity, right to self-determination, privacy and confidentiality of personal information of research subjects.” (WMA Declaration of Helsinki, 2013).

It has been tacitly assumed that the ethical guidance will be followed by the other non-medically qualified personnel. This strong relation of human research to the human health field and profession has been followed essentially unchallenged in the other ethical declarations, guidelines and international agreements dealing with human research published subsequent to the Nuremberg trial (The Belmont Report, 1979; The Oviedo Convention, 1997; Good Pharmaceutical Medical Practice, 2014; ICH Harmonised Guideline, 2016; International Ethical Guidelines for Health-related Research Involving Humans Final CIOMS, 2016). The ICH Harmonized Guideline refers only shortly to multidisciplinary research stating that “the investigator should maintain a list of appropriately qualified persons to whom the investigator has delegated significant trial-related duties.” In the CIOMS Guideline prepared in collaboration with the World Health Organization (WHO) it is only recommended that sponsors, researchers and research ethics committees “must ensure that all research personnel are qualified by virtue of their education and experience to perform competently and with integrity. This includes receiving appropriate ethics education and training. Qualifications of research personnel must be adequately described in the materials submitted to the research ethics committee.” Rapid scientific progress makes it, however, questionable whether this narrow approach is tenable, or the ethical issues should also be specifically addressed with inputs from other experts specifying also their ethical responsibilities.

Medical treatments became very sophisticated in recent years, many complex interventions can be performed only with the support of highly trained but non-medically qualified personnel. This is of great concern primarily for drug development groups which investigate for example advanced medicinal products such as gene and cell therapies, drug and medical device combinations. In such multidisciplinary teams the physicians work as team members, with special ethical responsibility to care for the well-being of the patients. Therefore, the physicians maintain a well-defined safeguarding role within the team, although he/she may no longer be in the position to understand the inputs of the various professionals in depth. Consequently, the physician of such teams cannot carry the entire ethical responsibility for the correct planning and conduct of the clinical trial alone. Inevitably the society has to decide whether the traditional clinician-centered ethical guidelines should be maintained or whether it is time to address the ethical responsibilities of the various non-medically qualified professionals directly involved as well.

The clinicians and non-medically qualified scientists have two main fields of interactions in the clinical development and application of medicines. The first occurs in translational medicine. The second contact is characterized by strong multidisciplinary cooperation in the development and therapeutic application of advanced therapies. As a result of the increasingly critical interaction of basic scientists with medical professionals many non-medically qualified scientists have become members of the IFAPP (2003). At present a large fraction of the IFAPP membership is not medically qualified, although pharmaceutical medicine was originally conceived as a medical discipline. It is a logical further step, that IFAPP decided to consider the ethical aspects of this collaboration and started to characterize the ethical responsibilities of the many non-clinicians involved in the research and clinical application of modern complex therapies. Supplementary Material: IFAPP International Ethics Framework for Pharmaceutical Physicians and Medicines Development Scientists, 2018.

Translational medicine provides a scientific bridge connecting non-clinical studies with the early exploratory evaluation of an investigational medicinal agent in humans (Littman et al., 2007). In reality, drug development gradually became part of an enlarged concept of pharmaceutical medicine. The safe and effective transfer of basic research results into the human research phase became a primary concern. New drug targets and biomarkers, the development of drug-medical device combinations, the methods of the preparation and administration of gene or cellular medicinal products are usually first investigated in animals by academic research groups. It is therefore very disappointing that from 53 landmark studies published in prestigious journals only 6% reported sufficiently robust data to drive reliably human medicines development programs (Begley and Ellis, 2012). Similarly, from 67 projects evaluated by a company 65% of the results published in the scientific literature could not be reproduced (Prinz et al., 2011). The broad experience of industrial R&D experts indicates that around 50% of findings published cannot be reproduced by the pharmaceutical industry (Booth, 2011). The inability of industry and clinical trial groups to reproduce the results of many academic publications on potential therapeutic targets and biomarkers suggests a general systemic problem, although occasional fraud cannot be ruled out.

Promising pre-clinical testing results frequently lead to rapid clinical development without thoroughly evaluating the quality of the data and the reproducibility of the experiments. This practice might lead, in unfortunate cases, to serious human suffering and wasting of valuable clinical resources. Superficially performed and/or interpreted animal-human translation studies might be considered one of the main components leading to system failures occurring in human phase I studies. Examples of two recent early clinical trial tragedies caused by TGN-1412 (TeGenero Immuno Therapeutics AG), (Reason, 2000; Suntharalingam et al., 2006; Sims, 2009; Attarwala, 2010), and by BIA 10-2474 (Bial-Portela & Ca. SA.) (Kerbrat et al., 2016; Greenberg et al., 2017) reminded the scientific community of the consequences for involved human beings. It became a main ethical requirement for effective and safe human drug development, that academic scientists should adopt research methods similar to those used in clinical trials to significantly improve construct validity of their research, especially the internal and external validities of the confirmatory pharmacotherapeutic studies in animals (Kilkenny et al., 2010; van der Worp et al., 2010; Arrowsmith, 2011; Kimmelman and London, 2011; de Vries et al., 2014; Kimmelman et al., 2014). With the translational concept animal and human studies gradually grow together to form a functional continuum. This bridge effectively binds experts of non-clinical research and clinical drug development into a functional continuum of partnership with shared ethical responsibilities. As a logical consequence it was considered necessary to include the ethical responsibilities of non-clinical researchers into the new revised version of the ethical framework of IFAPP.

Multidisciplinary teams gained broad acceptance in drug development when, beside the determination of clinical efficacy and safety, the correlations between the plasma level of the drugs and their pharmacodynamic effects also became the additional focus of clinical pharmacological investigations. Such cooperation is primarily characterized by the parallel work of the clinical and various non-medical experts who perform pharmacokinetic, biochemical, immunological and other investigations on human samples. The ethical problems of such cooperation are usually limited to the amount and frequency of the sampling of human materials needed for conducting the studies. The situations can be handled by finding a scientifically acceptable compromise which does not cause additional harm for the human subjects. A conceptually entirely different and much more sophisticated cooperation becomes necessary for investigating and applying advanced therapeutic products in patients.

The complexity of the scientific-medical approach can be convincingly demonstrated in the case of the recently developed Chimeric Antigen Receptor Adoptive T-cell (CAR-T) cancer therapy. For this treatment the genes coding for the specific CAR-T receptor recognizing the cancer surface antigen(s) of the individual patients must be transferred into the harvested T-cells of the patients. The modified T-cells are then further incubated *in vitro* before re-transfusion for reaching the required number of modified T-cells for effective tumor kill. The production of the individually prepared targeted medicinal product is carried out under Good Manufacturing Practice (GMP) conditions by a multidisciplinary expert team specialized in immunology, cell and molecular biology (Jacobson and Ritz, 2011; Yee, 2013; Sharpe and Mount, 2015; Hartmann et al., 2017). The therapy is a real team effort. The final therapeutic decisions must be made jointly by all the experts involved considering both the condition of the patient as well as the success and specificity of the CAR-T cell preparation to be used for the individualized therapy.

In such multidisciplinary teams the physician is only one member with a specific right to stop the intervention if the safety of the patient is endangered and the interruption of the therapy does not cause additional harm. It is not surprising that the FDA requires that the entire staff involved in this complex therapy should be specifically trained and certified (FDA News Release, 2017). The joint scientific-ethical responsibility of such a multidisciplinary team is obvious. Although it is assumed that all experts act according to the basic principles governing human research, respect for autonomy, beneficence, non-maleficence and justice (Beauchamp and Childress, 2012), different weighing of these principles might be expected under different conditions (Ebbesen and Pedersen, 2007; Page, 2012). Such differences must be resolved within the group for each case separately. To maintain successful cooperation, it must be ensured that each contributor is able to work according to the guiding principles of their professional organizations.

The rapid progress of advanced therapies will further increase the need for including many different professionals into clinical teams. In addition, new scientific knowledge continuously generates unforeseen ethical problems. For successfully managing increasingly sophisticated ethical challenges IFAPP recommends and plans to contribute to the strengthening education of ethics at the under-graduate and post-graduate levels both for medical and other biomedical professionals.

The aim of the linked IFAPP International Ethics Framework is to highlight the ethical issues relevant to the increasingly close cooperation of physicians and non-medically qualified experts in human drug development and application. Supplementary Material: IFAPP International Ethics Framework for Pharmaceutical Physicians and Medicines Development Scientists, 2018. The intention of the IFAPP Working Group on Ethics was to provide recommendations for supporting both medically and non-medically qualified investigators to make ethical decisions cooperatively under various, frequently unexpected, situations occurring during human drug research. We are convinced that the recommended joint decision-making process will be helpful for all scientists working all over the world in medicines development to find ethical answers to new challenges. It is also hoped that the revised edition of the IFAPP International Ethics Framework might be helpful for countries either to adjust their local recommendations to the new scientific environment or to introduce ethical guidance if not yet existent in their country.

## Author contributions

All authors contributed both to the development of the ideas as well as to the writing of the paper and the linked Supplementary Material: IFAPP International Ethics Framework for Pharmaceutical Physicians and Medicines Development Scientists (PPs & MDSs).

### Conflict of interest statement

The authors declare that the research was conducted in the absence of any commercial or financial relationships that could be construed as a potential conflict of interest.
