# Supplementary material for: The Shared Ethical Responsibility of Medically and Non-medically Qualified Experts in Human Drug Development Teams
Source: Front Pharmacol. 2018 Sep 3;9:843. doi: 10.3389/fphar.2018.00843 (PMC6130227; doi:10.3389/fphar.2018.00843)
Supplement: Supplementary file 1 [file Presentation_1.PDF]

International Federation of Associations of Pharmaceutical Physicians & Pharmaceutical Medicine

IFAPP INTERNATIONAL ETHICS FRAMEWORK FOR PHARMACEUTICAL PHYSICIANS AND MEDICINES DEVELOPMENT SCIENTISTS (PPs & MDSs*).

*** In this text MDS refers to experts in various fields of life sciences, pharmacy and medical device engineering who have adequate training in non-clinical and clinical medicines development and work as integrated members of clinical medicines development teams.**

2nd edition, March 2018

The 2nd edition, entitled IFAPP International Ethics Framework for Pharmaceutical Physicians and Medicines Development Scientists (PPs & MDSs*), was debated and developed by the IFAPP Working Group on Ethics in 2016: Drs. Sandor Kerpel-Fronius, chairperson (Hungary), Sander Becker, co-chairperson (Australia), Jane Barrett (UK), Johan Brun (Sweden), Roberto Carlesi (Italy), Anthony Chan (Ireland), Luis Collia (Argentina), Dominique Dubois (Belgium), Peter Kleist (Switzerland), Greg Koski (USA), Chieko Kurihara (Japan), Luis Filipe Laranjeira (Portugal), Johanna Schenk (Germany), and Honorio Silva (USA).

First edition published in April 2003

The IFAPP International Code of Ethical Conduct for Pharmaceutical Physicians was first created, debated and published by the IFAPP Working Party on Ethics in 2003: Drs Sander Becker, chairperson (Australia), Jane Barrett, co-chairperson (UK), Johan De Botha (S Africa), Johan Brun (Sweden), David Cairds (Germany), Anthony Chan (Ireland), Roberto Carlesi (Italy), Luis Collia (Argentina), Maqbool Jafary (Pakistan), Gerhard Nahler (Austria), Antti Jekunen (Finland), Francis de Halleux (Belgium), Ludger Buehrmann (Germany) and Alan Dunton (USA).

Content

PREAMBLE 3
1. Ethics Framework 4
Scientific progress creates new ethical problems for clinical drug development........................ 4
PRIMARY RESPONSIBILITY AREAS FOR PPs.. 6
2. Duty of clinical care 6
2.1 Good Clinical Practice 6
2.2 Medical integrity.. 7
JOINT RESPONSIBILITY AREAS FOR PPs AND MDSs.. 8
3. Competence and Diligence.. 8
3.1 General issues in clinical research 8
3.2 Studies of therapeutic use and post-marketing surveillance.. 9
3.3 Special and vulnerable patient groups 10
3.4 Benefit-risk assessments 11
3.5 Awareness of innovation 12
4. Impartiality in Communication of Information 13
4.1 Promotion.. 13
4.2 Provision of information.. 14
4.3 Information to health care professionals and competent authorities.............................. 14
4.4 Information to patients 14
4.5 Information to the media 15
5. Probity 15
5.1 Accurate reporting 15
5.2 Financial and commercial dealings.. 16
6. Integrity and Accountability in the Workplace.. 16
6.1 Propriety.. 16
6.2 Teamwork.. 16
6.3 Leadership 17
Conclusions and Outlook.. 17
Glossary and terms used in this document 19

PREAMBLE

The Ethics Framework, formerly known as the International Code of Ethical Conduct for Pharmaceutical Physicians,[1] has been updated considering the rapidly changing and increasingly complex scientific environment of medicines development.

The ethical behaviour of clinical drug researchers similar to other clinical researchers is based on internationally accepted ethical concepts, the Helsinki Declaration[2], CIOMS[3], Bellmont Report[4]. Nevertheless suggestions and recommendations for supporting ethical behavior within this framework is needed for the various medically and non-medically qualified participants of the modern multidisciplinary teams performing non-clinical and clinical research both during the development and life cycle management of medicnal products. Such guidance could also help the decision making of experts working in research ethics committes or hospital ethics committees which might have different influence on clinical reserach according to the local legal system. Each situation is different and under various circumstances the ethical decisions might also be different. Therefore, it was a principal decision of the members of the Working Group not to provide ethical guidelines or directives which were felt to be restrictive or imply disciplinary actions. Rather it is intended to advise both PPs and MDSs on possible ethical behavior listing some selected circumstances and appropriate actions.

PPs & MDSs are essential members of the teams working throughout the life-cycle of medicines and medical devices, from the discovery research phase, through pre-clinical and clinical testing, licensing, launching, post-marketing studies and surveillance to its eventual demise whether on grounds of relative safety and efficacy or commercial non-viability. Being critical members of a team there is an understandable tendency for PPs & MDSs to develop a strong belief in an intervention with which they have had a long or close association. Despite this, PPs & MDSs should recognize their ethical responsibility and stand aside from product loyalty when assessing factors affecting the product itself. They must remain aware at all times that the ultimate interests of both patients and their own employers are best served by an objective scientific attitude. The IFAPP recognizes that this may place practicing PPs & MDSs in a position which demands considerable determination. Ethics plays a vital role in enabling PPs & MDSs to reconcile their professional lives with their personal values.

Medical and scientific ethics do not yet feature prominently in the syllabuses of natural science and medical faculties of the universities throughout the world, yet it is increasingly recognized as important by regulatory bodies of medical practitioners and scientific societies. It follows that training in ethics in pharmaceutical medicine is itself important for all PPs & MDSs, health professionals, ethics committee members and other experts involved in the research and development of therapeutic agents. The IFAPP recommends that the education in ethics should

---

[1] IFAPP International Code of Ethical Conduct for Pharmaceutical Physicians. Published in 2003. http://ifapp.org/Ethics/Code-of-conduct

[2] WMA Declaration of Helsinki - Ethical Principles for Medical Research Involving Human Subjects http://www.wma.net/en/30publications/10policies/b3/

[3] Final CIOMS guidelines, 2016. CIOMS. www.cioms.ch/ethical guidelines-2016/

[4] The Belmont Report. http://www.fda.gov/ohrms/dockets/ac/05/briefing/2005-4178b_09_02_Belmont%20Report.pdf

feature in the various training courses provided for individuals seeking recognition either as PP or MDS. Through these efforts, the achievement of professional excellence can be fostered, and self-identity and professional aspirations supported.

The proposed Ethics Framework introduces the core values, then lists and discusses specific areas of ethical concern for PPs & MDSs to consider. It is our hope that the IFAPP International Ethics Framework, finalized after extensive worldwide consultation with National Member Associations of IFAPP and selected scientific Societies will be relevant for most parts of the world. In addition, it may stimulate those countries that have not yet adopted a framework of ethical conduct to consider such an approach. Only in this way can standards and values in pharmaceutical medicine become universally accepted. However, the recommendations of this Ethics Framework should not supersede the laws of a nation, except where local laws are less stringent.

1. Ethics Framework

Scientific progress creates new ethical problems for clinical drug development

The original IFAPP International Code of Ethical Conduct for PPs was published in 2003 with the fundamental assumption that pharmaceutical medicine and PPs across the globe embrace the following core values in the ethical discharge of their duties: the values of scientific responsibility, duty of care, competence and diligence, impartiality, probity, integrity and accountability in the workplace. Medical ethics have been shown to govern the human relationship between health care providers and patients as far as it can be traced back in history. However, as science becomes more innovative and medical research processes more creative, the ethical, cultural, ideological and demographical boundaries which were limited to “what was considered possible” are now being challenged by scientific and medical endeavors. Unfortunately, an alarming number of breakthrough scientific results fail when transferred to medical practice. In the future translational medicine must connect non-clinical and clinical drug development with much higher reliability than it is customary presently. For the further development of the efficacy and safety of pharmaceutical medical research it is essential that both the internal and external validities especially of the confirmatory animal pharmacotherapeutic studies should be significantly increased. To achieve these goals, it is recommended that the animal studies should accept stringent methodological standards in many respect similar to those applied in clinical trials. [5, 6, 7, 8, 9]

---

[5] Kimmelman J, Mogil, JS, Dirnagl, U. (2014) Distinguishing between Exploratory and Confirmatory Preclinical Research Will Improve Translation. PLoS Biol 12(5): e1001863. doi:10.1371/journal.pbio.1001863
[6] Begley CG and Lee M. Raise standards for preclinical cancer research
Ellis propose how methods, publications and incentives must change if patients are to benefit. Nature, 483: 531-533, 2012.
[7] Prinz F, Schlange T and Assadulah K. Believe it or not: how much can we rely on published data on potential drug targets? Nature Reviews Drug Discovery 10, 712, 2011| doi:10.1038/nrd3439-c1
[8] Arrowsmith J. Trial watch: Phase II failures 2008-2010. Nature Reviews Drug Discovery 10, 328-329. 2011. | doi:10.1038/nrd3439
[9] van der Worp HB, Howells DW, Emily S. Sena ES et al. Can Animal Models of Disease Reliably Inform Human

Nevertheless, the increasingly close cooperation between basic and applied medical researchers has led recently to some astonishing therapeutic successes. In particular, the concept of translational medicine necessitated the organization of medicines development teams including medical doctors and experts representing a large number of specialized basic research groups. Advanced therapies including gene and cell therapies, tissue engineering as well as sophisticated medical device and drug combinations requires the cooperation of non-medically qualified experts to enable proper administration of such treatments. As a consequence it became necessary to address the ethical responsibilities of the entire multidisciplinary team including both basic and medical research experts.

The bioethical principles of Beauchamp and Childress[10] – respect for autonomy, beneficence, non-maleficence and justice – play a determining role in the ethical behavior of both physicians and various research scientists working in pharmaceutical medicines research. They form a basis for balanced ethical judgement in conflict situations. Additional ethical principles of relevance include vulnerability, subsidiarity and solidarity, as well as ethical considerations of the duties to the society with regard to objective setting and appropriate research conduct. However, it has become evident that under various circumstances the different experts of medicines development put different weights to these principles. [11,12]

IFAPP believes that it has a responsibility to define and publish standards to which all parties involved in medicines development can refer. The need for such revision of the ethical principles is further underlined by the fact that a substantial percentage of the registered members of the International Federation of Associations of Pharmaceutical Physicians and Pharmaceutical Medicine (IFAPP) are medicines development scientists without a formal medical qualification. An International Working Group (WG) was established in 2015 to advise IFAPP how to manage the complex ethical issues arising within these highly specialized cooperative groups active in drug development and also during the entire life cycle of medicines. It is recognized that some ethical issues are of exclusive relevance for PPs or MDSs, respectively. The protection of the dignity, rights, needs and interests of the trial subjects will always remain the primary objective of the traeting physicians. However, most of the challenges have to be faced jointly by PPs and MDSs.

The IFAPP Ethics Framework intends to provide an educational background to guide both PPs and MDSs through their day-to-day deliberations and decision making irrespective whether they are practicing within a company, contract research organization, academic department and regulatory authority or work in ethics committees (Institutional Review Boards) or as independent consultants. All clinical development activities and medical support services must be provided by appropriately trained individuals working to agreed standards in adequately staffed departments having clear

---

Studies? PLoS Medicine | www.plosmedicine.org; March 2010 | Volume 7/Issue 3 |e1000245
http://journals.plos.org/plosmedicine/article/file?id=10.1371/journal.pmed.1000245&type=printable

[10]Beauchamp TL and Childress JF. Principles of Biomedical Ethics. Seventh Edition, 2012. ISBN: 9780199924585

[11] Ebbesen M and Pedersen BD. Empirical investigation of the ethical reasoning of physicians and molecular biologists – the importance of the four principles of biomedical ethics. Philosophy, Ethics, and Humanities in Medicine, 2:23, 2007. doi:10.1186/1747-5341-2-23

[12] Page K. The four principles: Can they be measured and do they predict ethical decision making? BMC Medical Ethics. 13:10, 2012. doi: 10.1186/1472-6939-13-10

responsibilities and the authority to take necessary decisions. Certification of PPs & MDSs with various academic backgrounds should be, in the future, an essential element of the demonstration of appropriate training in pharmaceutical medicine. Similar to all branches of medicine, certification of PPs & MDSs should not be seen as a single event but should be part of lifelong learning and subject to appropriate re-certification, as available.

PRIMARY RESPONSIBILITY AREAS FOR PPs

2. Duty of clinical care

2.1 Good Clinical Practice

- MDs and PPs are required, by the nature of their job, to follow ethical principles of medical practitioners, to keep themselves abreast of scientific advances that will have a major impact on the development of the new medicines of the future.
- A registered medical practitioner with appropriate specialist training should be in overall control of any research involving human subjects. This must include, as a minimum, basic training in clinical research methods, regulation of medicines development, medical ethics and up to date best practices.

Primary ethical framework areas for PPs:

- Maintaining high medical standards required by national and international regulations.
- Ensuring that they remain well informed about current scientific and medical knowledge in the areas of therapeutics in which they work.
- Assimilating constructive feedback from management, clical ethics committees and the regulatory authorities.
- Cooperating in the design of clinical research programs and protocols of high scientific quality and sound methodology in areas of medical need according to regulatory requirements, national and international codes of practice, and the Declaration of Helsinki.
- Ensuring that they fulfill their obligations in clarifying, evaluating, treating and reporting adverse events, whether they come from research protocols, spontaneous reports or as part of a formal surveillance program.
- Ensuring that prompt action is taken if the safety of the study subjects is being compromised.
- Ensuring that documents submitted to the regulatory authorities accurately reflect the data that have been gathered in the development process.
- Ensuring that information provided to health care providers is balanced and allows objective decision-making.
- Ensuring that all data of importance for the safety of patients and decision making are made available for publication and that articles submitted to journals accurately and completely reflect the data on which they are based.
- Provision of accurate and verifiable information to the company’s commercial department.
- Teaching, training, appraising and assessing of other members of the medical department.

2.2 Medical integrity

PPs have dual responsibilities, medical care and clinical research. Research involving healthy people or patients as study subjects must be justified by careful consideration of the risks and benefits involved. The health and well-being of each study subject is of paramount importance and relegates all other considerations to being of lesser importance. People volunteering to be study subjects, both healthy volunteers and patient volunteers, are required to give written informed consent after receiving sufficient and properly witnessed explanations of any potential risks and benefits involved. Particular care must be shown when studies include patients who are not volunteers and cannot give consent for themselves, whether due to their age (children) or lack of capacity (e.g., the unconscious or mentally incompetent). Financial compensation should be appropriate without constituting exploitation, coercion or bribery, and must be approved by the relevant ethics committees.

Specific ethical framework areas for PPs:

- Clarifying whether or not the company will provide continuation of support and interventions once a patient’s research involvement ends and the mechanism by which this will occur.
- Ensuring that the entry of a patient into a study with an investigational drug does not jeopardize his/her long-term care using the best available treatment.
- Ensuring that in the provision of disease management packages any conflict between competing interests is minimized, for example where the treatment of choice may be a therapeutic agent produced by a competitor.
- Ensuring that the best interests of individual patients always prevail over those of the employer and the society.
- Ensuring that information is provided in accordance with the principles of evidence-based medicine to optimize the acceptability of products for which they are responsible, but within appropriate clinical management guidelines.
- Resisting the use of therapeutic interventions outside of clinical management guidelines without conducting appropriate clinical trials and/or obtaining the necessary regulatory clearance.
- Utilizing individual clinical judgement over clinical management guidelines where it can be demonstrated that an alternative course of action is more appropriate for an individual patient.
- Reviewing local guidelines with regard to the payment to patients in clinical trials, and ensuring that local Ethics Committees are informed and consulted, and approval obtained if required.
- Ensuring that patients are not inappropriately induced to take part in clinical studies. Respecting their duty of care regarding the use of unlicensed or unproven interventions, regardless of the source.
- Assuming responsibility for any patients under their care, whilst the providers of the medial products carry responsibility for the quality of the supplied products.
- Ensuring that patient information leaflets are clear and fully understood by the end user.

JOINT RESPONSIBILITY AREAS FOR PPs AND MDSs

3. Competence and Diligence

3.1 General issues in clinical research

Intentions to perform research on humans must be carefully considered. The research team should follow international principles of research on human subjects as presented in documents such as the Helsinki Declaration, CIOMS, Belmont Report, etc. PPs and MDSs should jointly evalaute whether the non-clinical data are reliable and provide adequate basis for judging the expected safety and effectiveness of the proposed interventions. In addition they have to ensure that the scientific approach and methodology to be applied in the clinical research is current, the motivation is clear, the processes are unambiguous.

Specific areas of ethical concern for PPs & MDSs:

Research accountability

- Considering the scientific feasibility of translating animal data to humans
- Designing clinical study protocols to answer genuine scientific questions and minimize bias.
- Clinical studies should always be non-promotional in nature
- Implementing a clinical study protocol only after the approval of an independent ethics committee and the receipt of appropriate regulatory competent authorities approval.
- Protecting subjects as a priority over scientific interest in all study protocols, as outlined by the Declaration of Helsinki, CIOMS, Belmont Report.

General aspects

- Obtaining independent ethics committees approval for all clinical trials
- Ensuring that the study site chosen for a study, the principal investigator at the site and the entire support staff should be appropriately equipped and trained to properly care for each study subject and to perform the investigations at the required high quality standards at the planned times.
- Ensuring that scientific and ethical standards are constantly upheld.
- Resisting undue pressure on any investigator in order to meet deadlines.
- Ensuring that sufficient data are generated to allow the appropriate scientific evaluation of the safe and effective use of the investigational product.
- Ensuring that the trial subjects are appropriately informed and sign a specific consent form permitting or prohibiting the further use of their biological samples in specified or unspecified future scientific research.
- Ensuring that personal data are handled in compliance with data protection regulations.
- Ensuring that personal data of the research subjects are communicated to participating research laboratories and/or relevant external agencies only following proper anonymization or coding.
- Providing research ethics committees with all relevant information to enable them to make a considered judgement on the ethics of any given study protocol.
- Ensuring that payments for studies, details of the recipients, and potential conflicts of interest, are totally transparent to ethics committees.

- Ensuring that policies that safeguard the interests of study subjects, in terms of indemnity and compensation are in place.
- Realizing that it is unethical to change from an effective treatment to a trial medication unless there are sound scientific reasons for doing so, approval is obtained from a research ethics committee, the appropriate explanation is given to any trial subject and consent obtained.
- Remembering their duty to apply standards of scientific rigor and provision of quality information wherever they may be working, be it in research, production or marketing.
- Familiarizing themselves and the staff with the policy and standard operating procedure in place relating to the management of suspected fraud and demonstrating commitment for implementing the policy if occasion demands.
- Making a clear statement of the sponsor’s policy with regard to the handling of suspect data.

Clinical pharmacological studies

- Ensuring that comparison to placebo or to no treatment in clinical studies are performed only when it is scientifically unavoidable for reliably evaluating the efficacy and safety of an investigational product
- Minimize any risk of harm and/or additional discomfort due to investigational drugs for the patients.
- For safety reasons the approach of parallel administration of escalating doses of investigational drugs to several subjects for time saving must be avoided. Additional subjects should be treated only after the new dose was carefully evaluated in a single subject.
- Selecting appropriate medical, clinical laboratory and if needed basic science investigators prior to the start of a clinical trial, and ensuring that they are trained to appropriate levels.
- Ensuring that biological samples which can be obtained only by invasive methods should be collected only if the measurements are unavoidable for the proper scientific characterization of the investigational agent. The quantity of the biological sample should be the smallest amount required and the scientific methods applied should be of the highest reliable sensitivity available.
- Obtaining independent ethics committees approval which is needed also for microdose (phase 0) studies performed on humans.
- Ensuring volunteers recruited into human pharmacology or early exploratory phase clinical studies are provided with summaries of all important and relevant findings on the investigational agents.
- Ensuring that any volunteers recruited are not exploited, coerced or inappropriately remunerated.
- Ensuring no conflict of interest between those who design studies, including human pharmacology or early phase studies, and the teams responsible for their implementation and conduct.

3.2 Studies of therapeutic use and post-marketing surveillance

Once a medicinal agent has reached the stage where it is available for use, PPs & MDSs have an ethical responsibility to ensure that any studies they design, whether as therapeutic use trials, post-authorisation safety or efficacy studies or as non-interventional observational surveillance studies, will provide additional clinically relevant information regarding appropriate use of the new medicines in real life situations.

Specific areas of ethical concern:

- Ensuring that all studies have a well-defined scientific rationale.
- Ensuring that the handling of observational databases and extracting of interpretations from them be of the highest standard and do not violate personal rights of the subjects.
- Ensuring also that outcome results from Real-Word Data (RWD) trials are not assessed in a similar way as data from Randomized Clinical Trals (RCT) since real-world experience does not answer to a hypothesis stated but only tell us how things are not why.

3.3 Special and vulnerable patient groups

This group includes among others pregnant women, children, the elderly, the mentally incompetent, the terminally ill and the unconscious individuals. Under specific circumstances all people might become vulnerable and compromised, from prisoners, refugees to the impoverished, analphabetic patients etc. These groups should not be deliberately excluded from clinical studies as doing so may cause them and others in their condition more harm. Nevertheless, if they are to be included then very special circumstances apply and should be followed carefully. If such people are the intended beneficiaries of the interventions under study, sufficient information must be known about the interventions to make a risk versus benefit judgement possible. This usually means that at least some preliminary data from prior work on adults able to give informed consent will have been completed and should be available for information in the consent form.

3.3.1 Children

Specific areas of ethical concern:

- Ensuring that pediatric studies are only conducted where a therapeutic agent is intended for use in children.
- Ensuring that the local ethical position regarding the participation of children in research projects is clearly understood.
- Ensuring that informed consent should be obtained from parents or caretakers and assent be given by children considering their ability to communicate, weigh and understand the options which can vary with age.

3.3.2 Special risk groups

Specific areas of ethical concern:

- Ensuring that the local ethical position regarding the use of special risk groups (e.g., the elderly, the mentally incompetent, those with terminal or life threatening diseases, those who are socially or economically disadvantaged, or any condition or circumstance where reliable informed consent may not be obtained) is clearly understood by those organizing and conducting the clinical trial
- Clearly understanding the local ethical position regarding non-therapeutic trials without direct benefit to the patients involved (e.g., pharmacokinetic studies in subjects with hepatic or renal impairment).

3.3.3 Research in developing countries

The locally prevailing social attitudes must be carefully considered when contemplating studies using volunteers from developing areas of the world. Judgements of what is appropriate will vary according to social, ethical, economical and governmental factors, which are local and not necessarily international. The principles of beneficence, non-maleficence and respect for human dignity prevail over any other consideration.

Specific areas of ethical concern:

- Ensuring that special care is taken to obtain local independent ethical review and approval if needed with the support of international, independent advisers, where the approval of an ethics committee in a developed country is not sufficient or relevant.
- Consider the ethnic, social, public health and economic conditions prevailing in the country concerned when any form of clinical research is being considered.
- Ensuring that low level of local health care and/or health care emergencies are not misused to overrule generally accepted ethical norms in conducting clinical trials.
- Avoiding the use of placebo controls in conditions where such controls would not be acceptable in the developed world.

3.3.4 Rare diseases and orphan medicines

A particular ethical dilemma arises with regard to rare indications and orphan therapeutic interventions, i.e., where too few patients have a disease for a treatment to be fully investigated in the usual way. There are some rare conditions for which it is clear that there will never be a viable commercial return on investment. In such circumstances the intervention may also not be assessable using the normal criteria.

Specific areas of ethical concern:

- Making the limited information available to physicians about orphan indications or rare conditions, such that they can give advice to patients or their families.
- Critically assess and ethically determine who might get access to orphan medicines if recommended by the treating physician.
- Providing scientific support for developing the most acceptable rationale for applying orphan drugs with limited available information in special cases.
- Providing information on suitable biomarkers if available for selecting the right patients for treatment.
- Getting critically involved in the concept of adaptive licensing, provide scientific and if possible clinical input/support for determining the appropriate time to allow treating patients with experimental products which lack conclusive scientific data needed from a regulatory perspective.

3.4 Benefit-risk assessments

If the PPs & MDSs are not certain about all aspects of the status of a clinical research program, even in the light of acceptable efficacy or safety, it is appropriate to delay making a decision on its future

progress until any doubts have been resolved. Making an inappropriate decision before such concerns are resolved is unethical. Thus, both when assessing the outcome of a clinical trial program and when reviewing the safety profile after marketing, PPs & MDSs must actively fulfill their scientific and ethical responsibilities.

Specific areas of ethical concern:

- Ensuring that no early decisions are made on either efficacy or safety before minimal amount of sufficient data are available.
- Ensuring that adequate systems are in place to ensure the timely capture and analysis of relevant data upon which a decision to withdraw or modify an intervention might be based.
- Balancing the potential benefit for the majority against the possible harm done to a minority of the trial subjects.
- Ensuring, in the case of a withdrawal of any therapeutic intervention, that as much relevant information as possible is made available to enable the clinical care of patients who are affected by the withdrawal to continue therapy with minimal disturbance.
- Appreciating that it is ethical to stop further patient recruitment into a trial when the observed burden of adverse reactions outweighs the expected medical and/or scientific benefit of a trial.
- Appreciating that it is ethically imperative to make new medical products available for patients with life-threatening diseases as soon as practicable when efficacy is unequivocally proven but before benefit-risk evaluation is completed.
- Appreciating the scientific and ethical need for the continuous collection of follow-up data of medical interventions marketed on the basis of adaptive pathways regulation.
- Ensuring that the collection of patient reported outcomes and real life data on newly marketed medical products is pursued with scientific rigor and ethical principles generally accepted for clinical development of medical products.
- Ensuring that the primary focus of PPs and MDSs participating in health technology assessment, price and reimbursement policy making is guided by scientific facts and the medical needs of the patients.

3.5 Awareness of innovation

It is essential that PPs & MDSs maintain awareness of new technologies and therapeutic approaches in the wider context of the practice of medicine where new ethical issues might arise. Such interventions include for example the sampling and use of human body parts and products including organs, tissues, fluids or gametes, advanced therapies, interface with medical devices and delivery mechanisms. Especially studies with in-vitro fertilization, or other methods of medically assisted procreation where medical products may be involved, prenatal diagnosis, certain aspects of contraception and abortion might lead to major ethical and political controversies within society.

Specific areas of ethical concern:

- Understanding and respecting local regulations and international conventions governing the collection, management and specific analysis of clinical data for which consent may not necessarily have been given.
- Ensuring the maintenance of high ethical standards and fulfillment of appropriate legal requirements when using electronic clinical data management.

- Protecting the personal rights of the patients when designing study protocols using pharmacogenetic profile, specific molecular targets, biomarkers, etc. for selecting and classifying patients.
- Ensuring that advanced therapeutic materials such as gene products, cells, tissues, etc. which are frequently specifically produced for the individual patients by basic research members of the clinical team should be prepared according to the quality standards accepted in the development of medicines.
- Ensuring that measurements of new biomarkers not yet validated or routinely used in clinical practice should be appropriately standardized and applied with strict quality control according to the principles of Good Clinical Laboratory Practice.
- Appreciating that the results of biomarker investigations should be considered for clinical decision-making only after proper validation and thorough understanding of their clinical relevance.
- Applying research data from breakthrough basic science experiments directly for planning clinical trials needs additional critical scrutiny regarding their internal and external validities both by the ethics committees and the applying clinical teams.
- Ensuring that the molecular endpoints of clinical trials selected from laboratory research data have sufficient construct validity and pathophysiological plausibility.
- Making sure that the personal rights of the patients in clinical trials are carefully protected by adequate anonymization or coding of their biological samples, especially biopsies, before transmitting them to various research teams involved in the trial or to other external organizations.
- Considering the ethical implications and consequences for the patients and their families of using genetic data.

4. Impartiality in Communication of Information

Non-clinical and clinical studies are performed to improve scientific knowledge, and this knowledge should be shared with the wider world. Study findings should be communicated irrespective of outcome (positive or negative) for the benefit of the community at large. Communications on both non-clinical and clinical studies must be an unbiased representation of all findings, allowing others in turn to derive their own interpretations and conclusions, so as to provide well-balanced advice to patients and their families.

4.1 Promotion

Specific areas of ethical concern:

- Ensuring promotional claims made are considered justified and supported by scientific evidence.
- Ensuring that any promotional material or activity does not contravene the advertising regulations and codes of practice of the countries concerned.
- Scientific communication should be clearly distinguished from promotional activity. Market research and promotional activities should not be disguised as scientific research.

4.2 Provision of information

Specific areas of ethical concern:

- Ensuring that information provided to doctors, pharmacists, patients and members of the public is appropriately phrased for the specific audience and is scientifically accurate.
- Provide adequate information that products are used appropriately in the countries where they are placed on the market.
- Recognizing that summaries of product characteristics exist not only to fulfill legal requirements, but also to help physicians to use products safely and correctly.

4.3 Information to health care professionals and competent authorities

Effective and safe use of medical products,- (licensed or unlicensed) - depend on the access to data published or unpublished which are necessary for the physicians to make rational therapeutic decisions. Doctors are increasingly being encouraged to practice medicine based on all the available evidence in an attempt to further improve quality of health care. It is recognized that physicians sometimes prescribe medicines for indications or in dosage regimens that are not in accordance with the terms of the product's marketing authorization. Where off-label use is permitted it should be based on scientific considerations. To this end it can be appropriate to share with prescribing doctors upon request data that are not yet in the public domain.

Specific areas of ethical concern:

- Compliance with local legislations relating to clinical trial registration and information disclosure.
- Compliance with local regulation limiting communication about product information under clinical trial
- The principle to make public clinical trial results as soon as possible after completion of the clinical studies should be encouraged.
- PPs & MDSs have a particular ethical responsibility to ensure that all the evidence upon which treating physicians base their decisions are easily available.
- Ensuring that data to support marketing position statements should be of the same high quality, and conform to the same scientific criteria, regardless of whether they are published or unpublished.
- Encouraging the provision of all information known about an intervention published or unpublished to physicians requesting it for individualized treatment of patients needing special care with medical products not yet authorized in a given indication.
- Informing the physicians to advise their patients that the applied intervention is not yet licensed for use in the disease to be treated.
- Ensuring that all adverse events observed during off-label application of medical products should be reported to the competent authorities.

4.4 Information to patients

Encourage patient to seek as much information as they wish.

Specific areas of ethical concern:

- Ensuring that there is a clear understanding of the difference between providing clear information to patients and the offering of advice.
- Personal advice about a specific patient's treatment should be given only via the treating physician.
- Ensure that information intended for patients is appropriately directed and suitably written so patients and their families are able to make an informed decision about their treatment and medication.
- Ensure when the applied medical product is not yet licensed for use in the disease to be treated, then the patient obtains adequate medical and access information according to local legislation
- Ensuring that non-medically qualified members of a clinical development group do not influence patients and/or the public with scientific results without embedding these results into appropriate medical context.

4.5 Information to the media

Specific areas of ethical concern:

- Ensuring that expectations are not inappropriately raised as a result of the release of media briefings. All such materials should be non-promotional in nature.
- Reviewing briefings about potential therapeutic interventions provided to financial analysts or to the media. All such materials should be non-promotional in nature.
- Drawing to the attention of the authorities on misleading information of any medicine, for example via the Internet and websites which may violate national legislation.

5. Probity

PPs & MDSs frequently work directly or indirectly for commercial organizations. They must, therefore, be extra vigilant that their decisions and practices are not in any way influenced by any personal financial gain. Each of the many groups and individuals involved in planning, sponsoring and performing such studies must always declare potential conflicts of interest that might influence the making of balanced, unbiased judgements relating to the medical products under discussion.

5.1 Accurate reporting

Specific areas of ethical concern:

- Ensuring that final study reports accurately reflect the study protocol, the pre-specified statistical analyses, and the obtained clinical data. Any publication that is derived from the data should be balanced and wholly consistent with the report.
- Maintaining the principle that reports should be published regardless of their impact on the medical product or its manufacturer.
- Ensuring that all studies performed are analyzed and have a report written, however brief, to ensure that no valuable data will be lost, and to allow for continuous risk-benefit evaluation. This information should, wherever possible, lead to publication or at least should become accessible on request by concerned parties.

- Ensuring that promotional materials follow appropriate national approval regulations and only contain substantiated claims.
- Balancing the need to make promotional material interesting and attractive against the need for scientific and medical accuracy.
- Ensuring that the commercial interest of a company is never allowed to take precedence over the requirement to report all safety data and adverse drug reactions to the respective competent authorities.

5.2 Financial and commercial dealings

Specific areas of ethical concern:

- Refusal to provide or accept gifts or hospitality designed to influence professional judgement.
- Compensation for carrying out a clinical trial must be commensurate with the work required and not structured in such a way as to encourage coercive behavior.
- Declaration of financial interests in clinical development, dealings with colleagues, the editors of scientific journals and the general public.
- Payments for any reasons, to both volunteers and those performing the study must not provide undue incentives and must be transparent and known to the Ethics Committees as required, as must any potentially conflicting interests.
- Arrangements for product liability, indemnity and compensation in the event of anyone suffering harm must be clear, both to Ethics Committees and potential study subjects.

6. Integrity and Accountability in the Workplace

6.1 Propriety

Specific areas of ethical concern:

- Ensuring that colleagues are always treated fairly and are not discriminated against in any way.
- Ensuring that a colleague's lifestyle, culture, beliefs, color, gender, sexuality, or age does not prejudice a professional relationship with them.
- Ensuring that subjects' trust in the care or treatment they receive, or in the judgement of those treating them, is not undermined by the malicious or unfounded criticisms of colleagues.

6.2 Teamwork

Multidisciplinary teams play a rapidly increasing role in pharmaceutical research. Working in a team does not abdicate personal accountability for professional behaviors and the care provided. Recently patient groups were also included as active participants into the development of medicines, adding their special experience as appropriate.

Specific areas of ethical concern:

- Maintaining the integrity of a multidisciplinary clinical team by respecting the skills and contributions of colleagues with different expertise.

- Providing a climate for productive cooperation, open discussion and joint decision-making without jeopardizing the right of the physician in charge to intervene if needed for securing the well-being of trial subjects.
- The cooperation of patient groups in medicines development requires full understanding of their role within the development teams
- Effective communication of development related issues with colleagues within and outside the team.
- Ensuring that all data necessary for the reliable evaluation and interpretation of the trial results are made available to all members of the team and if necessary to experts outside the team.
- Participation in regular reviews and audits of the standards and performance of the team.
- Willingness to deal openly and supportively with problems in the performance, conduct or health of team members.

6.3 Leadership

Specific areas of ethical concern:

- Ensuring that all members of multidiscilinary clinical teams meet the appropriate standards of professional conduct and care.
- Arranging for the provision of sponsor physician availability at all times during an ongoing trial to answer any questions from investigators.
- Addressing any problems that might prevent colleagues from other professions following guidance from their own regulatory bodies.
- Ensuring that all team members understand their personal and collective responsibility for the safety of patients as well as the scientific validity of the research.
- Ensuring a collaborative environment for open and honest discussion of issues.
- Ensuring regular reviews and audits of the standards and performance of the team and addressing any deficiencies.
- Ensuring that systems are in place for dealing supportively with problems in the performance, conduct or health of team members.

Conclusions and Outlook

This comprehensive list of recommendations compiled by two successive IFAPP Working Groups on Ethics tried to provide some practical recommendations how to behave under circumstances which might be faced by the teams or individual team members, when participating in clinical development of medical product and/or during their subsequent life-cycle management.

This Framework cannot cover all the situations which might occur during the very complex work of medical product management. It attempts to cover at least some of the new ethical problems which surfaced with the introduction of new scientific approaches. The discipline is constantly evolving and the working group will continue to evaluate if new guidances may become appropriate. All scientists involved in the development of medical products devices are invited to help us to extend

appropriate recommendations. Finally, it is also our strong hope that the IFAPP Ethics Framework will be integrated into worldwide teaching materials for educating future PPs and MDSs on proper ethical behavior.

Glossary and terms used in this document

- **Advanced-therapy medicinal products (ATMPs):** are medicines for human use that are based on genes or cells: gene therapy medicines; somatic-cell therapy medicines; tissue-engineered medicines; combined ATMPs
- **Basic scientists:** the term as used in this document refers to experts who are not medically qualified, work in various fields of natural sciences, pharmacy and medical device engineering with the primary aim to develop scientific knowledge and predictions
- **Drug:** is defined as a substance recognized by an official pharmacopoeia or formulary.
  - A substance intended for use in the diagnosis, cure, mitigation, treatment, or prevention of disease.
  - A substance (other than food) intended to affect the structure or any function of the body.
  - A substance intended for use as a component of a medicine but not a device or a component, part or accessory of a device.
  - Biological products are included within this definition and are generally covered by the same laws and regulations, but differences exist regarding their manufacturing processes (chemical process versus biological process.)
  - FDA Glossary: http://www.fda.gov/Drugs/InformationOnDrugs/ucm079436.htm
  - (The expressions drug and medicine and medicinal product are used as synonyms in this document)
- **Ethics committee:** the expression used in this document refer to all types of ethics committees dealing with clinical research and treatment of human subjects (research ethics committee, hospital ethics committee, Institutional Review Board, etc.)
- **Ethics Framework:** this expression was used for emphasizing the intention of the Working Group on Ethics to provide a collection of advices on possible ethical behavior listing some circumstances and appropriate actions instead of strict guidelines.
- **Impartiality:** absence of favor or prejudice (synonym: fair)
- **IFAPP:** International Federation of Associations of Pharmaceutical Physicians
- **Medical Device:**

  According to the FDA definition means. … an instrument, apparatus, implement, machine, contrivance, implant, in vitro reagent, or other similar or related article, including any component, part, or accessory, which is
  - recognized in the official National Formulary, or the United States Pharmacopeia, or any supplement to them,
  - intended for use in the diagnosis of disease or other conditions, or in the cure, mitigation, treatment, or prevention of disease, in man or other animals, or
  - intended to affect the structure or any function of the body of man or other animals, and which does not achieve its primary intended purposes through chemical action within or on the body of man or other animals and which is not dependent upon being metabolized for the achievement of its primary intended purposes
  - http://www.fda.gov/regulatoryinformation/guidances/ucm258946.htm

  According to the EMA definition means any instrument, apparatus, appliance, software, material or other article, whether used alone or in combination, including the software intended by its manufacturer to be used specifically for diagnostic and/or therapeutic purposes and necessary for its proper application, intended by the manufacturer to be used for human beings for the purpose of:
  - diagnosis, prevention, monitoring, treatment or alleviation of disease,
  - diagnosis, monitoring, treatment, alleviation of or compensation for an injury or handicap,
  - investigation, replacement or modification of the anatomy or of a physiological process,
  - control of conception,
  - and which does not achieve its principal intended action in or on the human body by pharmacological, immunological or metabolic means, but which may be assisted in its function by such means;
  - http://eur-lex.europa.eu/legal-content/EN/TXT/?uri=CELEX:01993L0042-20071011.htm
- **Medical product:** in this document medical products refers to drugs, medicinal products and medicines, biologics, vaccines, medical devices, nutriceuticals, diagnostics, cosmetics and their combinations used in medical practice.
- **Medicinal product, medicine** is defined as:
  - (a) Any substance or combination of substances presented as having properties for treating or preventing disease in human beings; or

- (b) Any substance or combination of substances which may be used in or administered to human beings either with a view to restoring, correcting or modifying physiological functions by exerting a pharmacological, immunological or metabolic action, or to making a medical diagnosis.’
